# 基于代谢组学探究表观遗传调控对老鼠簕内生真菌抗卵巢癌代谢物的影响

**DOI:** 10.3724/SP.J.1123.2024.08002

**Published:** 2024-11-08

**Authors:** Xiaolin MA, Laiyan CAI, Yanying LIU, Shangping XING, Liang KANG, Xia WEI, Dan ZHU

**Affiliations:** 1.广西医科大学药学院, 广西 南宁 530021; 1. Pharmaceutical College, Guangxi Medical University, Nanning 530021, China; 2.广西生物活性分子研究与评价重点实验室, 广西 南宁 530021; 2. Guangxi Key Laboratory of Bioactive Molecules Research and Evaluation, Nanning 530021, China; 3.广西医科大学第二附属医院药学部, 广西 南宁 530007; 3. Department of Pharmacy, the Second Affiliated Hospital, Guangxi Medical University, Nanning 530007, China

**Keywords:** 液相色谱-质谱, 内生真菌, 抗卵巢癌活性, 表观遗传修饰, 代谢组学, liquid chromatography-mass spectrometry (LC-MS), endophytic fungi, anti-ovarian cancer activity, epigenetic modifications, metabolomics

## Abstract

本研究旨在探究表观遗传修饰策略对老鼠簕内生真菌*Diaporthe goulteri*产生抗卵巢癌代谢物的影响。研究以抗卵巢癌活性为指标,采用CCK-8(cell counting kit-8)方法筛选粗提物的活性,控制发酵时间、修饰剂种类和浓度等变量,进而采用超高效液相色谱-质谱联用方法进行非靶向代谢组学分析,通过主成分分析和正交偏最小二乘判别分析,构建了多变量统计分析模型,结合模型和变量重要性投影(VIP),使用Massbank数据库进行定性,筛选并鉴定出15种在控制组与修饰组之间具有显著性差异的代谢物(VIP≥1, *P*<0. 05),主要包括聚酮类、氨基酸及衍生物类、生物碱类与有机酸类化合物等,其中10种物质含量上调,5种物质含量下调。研究结合了抗卵巢癌活性筛选与代谢组学分析,旨在发现潜在的活性代谢物,为红树植物老鼠簕资源的深入开发和活性化合物的靶向分离提供了科学依据。

## Abstract (en)

Ovarian cancer is a serious threat to women’s health and safety. So far, people have discovered more than 130 small molecule compounds of natural origin for anti-tumor, of which approximately 50% are of microbial origin. The *Acanthus ilicifolius* L. species is primarily distributed in the Guangdong, Hainan, and Guangxi regions of China and grows in tidally accessible coastal areas. Recent studies have revealed that *Acanthus ilicifolius* L. extracts are endowed with a range of pharmacological properties, including anti-inflammatory, hepatoprotective, antioxidant, and antitumor activities. Endophytic fungi are commonly found in the healthy tissue and organs of medicinal plants. These fungi and the plants they inhabit form mutually beneficial symbiotic relationships. Endophytic fungi produce a series of secondary metabolites, with active substances having shown great economic value and applications prospects in drug research and development as well as for the biological control of plant diseases. Secondary metabolites production by endophytic fungi is regulated by specific gene clusters, and several techniques have been used to stimulate the secondary metabolic processes of fungi, including epigenetic-modification and OSMAC (one strain many compounds) strategies, co-culturing, and gene modification. Among these, epigenetic modification has been shown to be effective; this strategy involves the addition of small-molecule epigenetic modifiers to the culture medium, thereby activating silenced biosynthetic gene clusters without altering the DNA sequences of the fungi. This approach facilitates the expression of silenced genes in endophytic fungi, thereby increasing the number and diversity of secondary metabolites. Furthermore, it assists in overcoming the inhibition of microbial secondary-metabolite synthesis under laboratory conditions, and enhances silenced-gene expressions.

The advent of novel analytical techniques and bioinformatics has provided a comprehensive, multifaceted, and holistic understanding of fungal metabolism through the development of metabolomics as a research platform. However, few studies have combined anti-ovarian cancer-activity screening with metabolomic approaches in the search for activity-differentiating metabolites from endophytic fungi under the intervention of epigenetic modifiers.

Herein, we investigated the impact of epigenetic modifiers on the secondary metabolites of the endophytic *Diaporthe goulteri* fungus from *Acanthus ilicifolius* L. to determine their potential anti-ovarian cancer activities. Crude extracts were obtained by controlling three variables: the number of fermentation days, the type of epigenetic modifier, and its concentration, with activities screened using the CCK-8 (cell counting kit-8) method. Ultra performance liquid chromatography-tandem mass spectrometry (UPLC-MS/MS) was subsequently employed for non-targeted metabolomic analysis. A multivariate statistical analysis model was constructed using principal component analysis and orthogonal partial least squares-discriminant analysis, which combines model and variable importance projection, with qualitative screening performed and significant changes (variable importance in the projection (VIP)≥1; *P*<0. 05) determined. Fifteen differential metabolites were identified in the fungal and epigenetic modification group, primarily comprising polyketides, amino acids, derivatives, alkaloids, and organic acids, including prenderol, glycine, valine, 2-ethylcaproic acid, rubratoxin B, finasteride, 6-silaspiro[5.5]undecane, 1-(2-nitrophenoxy)octane, heptadecene, 1-pentadecene, 11-ketoetiocholanolone, 3-(1-ethyl-1,3,3-trimethyl-2,3-dihydro-1*H*-inden-5-yl)butanal, *N*^2^-benzoylarginine, tabutrex, (3a*R*,6*S*,6a*S*)-6-(4-hydroxy-2-methoxy-2-butanyl)-4,4-dimethylhexahydro-1(2*H*)-pentalenone, and 8-aminoquinoline. The expressions of prenderol, 1-(2-nitrophenoxy)octane, 3-(1-ethyl-1,3,3-trimethyl-2,3-dihydro-1*H*-inden-5-yl)butanal, *N*^2^-benzoylarginine, and 8-aminoquinoline were downregulated, whereas the expressions of the remaining 10 substances were upregulated. Polyketides were the main components that exhibited higher expressions. This study showed that latent active differential metabolites can be searched by combining anti-ovarian cancer-activity screening with metabolomics analysis, thereby providing a reference for the further development of *Acanthus ilicifolius* L. resources and the subsequent targeted isolation of active compounds.

卵巢癌(ovarian cancer)是常见的女性生殖系统肿瘤,发病率仅低于乳腺癌和肺癌,在妇科恶性肿瘤中名列第三^[1]^。但其死亡率位居妇科恶性肿瘤的首位,5年生存率一直徘徊在30%~40%范围内,严重威胁妇女健康和生命安全。截至目前,人类发现天然来源的抗肿瘤的小分子化合物有130余种,其中约50%的化合物为微生物来源^[2]^。

老鼠簕(*Acanthus ilicifolius* L.)是爵床科老鼠簕属红树植物,具有清热解毒、消肿散结、止咳平喘的功效,主要分布在中国的广东、海南和广西等地区,生长在海岸及潮汐可达的滨海地带^[3]^。近年来的研究发现,老鼠簕提取物具有抗炎、保肝、抗氧化、抗肿瘤等多种药理作用^[4]^。药用植物的健康组织和器官中普遍存在着内生真菌,其与植物之间存在互惠互利的共生关系,且内生真菌能够产生一系列次生代谢物,这些活性物质在药物研发、植物病害生物防治等方面表现出了巨大的经济价值及应用前景。

内生真菌在常规实验培养条件下通常处于沉默或低表达的状态,获得次级代谢产物的量也往往较低。目前,已有多种方法被用于刺激真菌的次级代谢过程,例如表观遗传修饰策略^[5]^、单菌多次级代谢产物(one strain many compounds, OSMAC)策略^[6]^、共培养^[7]^及基因修饰^[8]^等,其中,表观遗传修饰策略是一种有效的方法,它通过在培养基中添加小分子表观遗传修饰剂来激活沉默的生物合成基因簇,而不改变真菌的DNA序列^[9]^,提高沉默基因的表达水平^[10]^。常用的有两种化学表观遗传修饰剂,即DNA甲基转移酶抑制剂和组蛋白去乙酰化酶抑制剂。

随着分析技术和生物信息学领域的进步,代谢组学作为研究工具,为我们全面、多角度地探索真菌的代谢过程及深入理解其代谢机制提供了新的视角^[11]^。代谢组学主要研究生物体及其组织中1000 Da以下的小分子代谢物,解释在特定时间、条件下某生物体内所构建的代谢网络,全面综合地分析某一生物体内部代谢状况,揭示生物体功能^[12⇓⇓⇓⇓-17]^。如牟红梅等^[15]^采用超高效液相色谱-串联质谱(UPLC-MS/MS)非靶向代谢组学技术,探究西洋梨品种茄梨及其红色芽变红茄梨成熟期果皮的代谢产物差异,筛选并鉴定出差异代谢物83种,主要包括酚酸类和黄酮类物质。王冀菲等^[16]^研究Pb^2+^胁迫对大麦籽粒中差异代谢物及关键通路的影响,采用液相色谱-质谱联用代谢组学技术,筛选出50种差异代谢物。马占君等^[17]^采用液相色谱-串联质谱技术,对结肠癌临床血清样本中胆固醇及相关10种氧固醇代谢物进行了定性定量分析,筛选得到3种代谢标志物。然而,结合抗卵巢癌活性筛选与代谢组学方法寻找内生真菌在表观遗传修饰剂干预下的活性差异代谢物的报道尚少。

本研究以抗卵巢癌活性为导向,采用UPLC-MS/MS技术对经过表观遗传修饰剂干预前后的内生真菌次级代谢产物进行非靶向代谢组学分析,寻找潜在的活性差异代谢物,以探究表观遗传修饰剂对老鼠簕内生真菌次级代谢的影响,为后续分离老鼠簕内生真菌中抗卵巢癌活性成分提供了理论和实验依据。

## 1 实验部分

### 1.1 仪器、试剂与材料

UPLC-Xevo G2-XS QTof超高效液相色谱-质谱仪(Waters公司,美国); XS205DU十万分之一电子天平(Mettler Toledo公司,瑞士); BL-50A立式压力蒸汽灭菌器筒(上海东亚压力容器制造有限公司); ACB-4A1超净工作台(艺思高科技有限公司,新加坡); ZDR-5210自动新型鼓风干燥箱(上海智城分析仪器制造有限公司); RE-5210A旋转蒸发器(上海亚荣生化仪器厂)。

5-氮杂胞苷(5-aza,≥99%)和伏立诺他(SAHA, *N*-羟基-*N*'-苯基辛二酰胺,≥99%)购自上海吉至生化科技有限公司;乙酸乙酯(分析纯)购自成都市科隆化工试剂厂;葡萄糖、琼脂购自北京索莱宝科技有限公司;甲醇(质谱级)购自美国TEDIA公司;乙腈(质谱级)购自美国Thermo Fisher公司;甲酸(质谱级)购自天津市科密欧化学试剂有限公司;二甲基亚砜(DMSO,细胞纯)购自美国MCE公司;胎牛血清和1640培养基购自美国Gibco公司;CCK-8(cell counting kit-8)试剂盒购自上海雅酶生物医药科技有限公司。

菌株*Diaporthe goulteri*分离自红树植物老鼠簕。植物样本采摘于广西合浦县山口红树林自然保护区,菌株由课题组前期从老鼠簕根中分离得到,编号为AIL-R-03,菌种保存在广西医科大学药学院。卵巢癌细胞SKOV3由广西医科大学药学实验中心惠赠。

### 1.2 实验方法

#### 1.2.1 样品发酵

在500 mL锥形瓶中加入70 g大米和110 mL纯水,制作大米培养基,修饰组选用最常用的DNA甲基转移酶抑制剂SAHA与组蛋白去乙酰化酶抑制剂5-aza,将SAHA与5-aza分别加入培养基中,使终浓度均为100、300、500、800、1000 μmol/L,对照组不加表观遗传修饰剂。120 ℃高压灭菌30 min,取出放凉。在净化工作台中操作,每瓶加入7~8 mL菌液,封口后取出,室温避光静置不同天数(7、14、21、28、35、42、49 d)。每组3瓶,共发酵252瓶。

发酵完成后,加入200 mL乙酸乙酯超声浸泡6 h,浸泡3次后合并提取液,经过滤、萃取、减压浓缩后,初步得到粗提物。加入50 mL蒸馏水溶解粗提物,随后用50 mL石油醚萃取脱脂,弃去石油醚层,接着在水层中添加50 mL乙酸乙酯萃取液体至无色,合并有机相,减压浓缩得到84个粗提物样品。

#### 1.2.2 CCK-8实验

精确称取每个粗提物样品2 mg,分别加入20 μL二甲基亚砜,超声溶解,经0.22 μm有机滤膜过滤后,配制成终浓度为0.1 mg/μL的母液。使用时用完全培养基(1640培养基∶血清=9∶1,体积比)稀释,体系中二甲基亚砜终浓度(体积分数)不超过0.1%。

采用CCK-8法检测样品对卵巢癌细胞SKOV3的抗肿瘤活性。按每孔3000个细胞加入96孔板,分为药物组与不加药物的对照组,每组设置3个复孔,于培养箱中培养24 h至贴壁完全。将药物用完全培养基稀释到预定浓度,弃去原96孔板中的培养液,加入稀释好的药物,每孔100 μL。给药后于37 ℃、5% CO_2_培养箱中培养48 h。药物作用48 h后,在避光条件下,以CCK-8试剂与1640培养基以1∶9的体积比配制工作液,将原有的培养基和药物吸出并丢弃,每孔加入工作液100 μL,于37 ℃、5% CO_2_的孵育箱中孵育1 h左右,采用酶标仪检测450 nm波长处的光密度(optical density, OD)值。根据以下公式计算细胞存活率:细胞存活率=(OD_药物组_-OD_空白组_)/(OD_对照组_-OD_空白组_)×100%。

#### 1.2.3 样品获取与制备

修饰组加入100 μmol/L SAHA,对照组不加表观遗传修饰剂,室温避光静置7 d。每组6瓶,共发酵12瓶。发酵与提取方法与1.2.1节相同,共得到12个粗提物样品。

将12个粗提物样品溶于质谱级甲醇中配制成500 ng/mL供试品,经0.22 μm微孔滤膜置于样品小瓶内,备用。质量控制(quality control, QC)样品为两组的等体积比混合样品。整体实验流程图见图1。

**图1:**
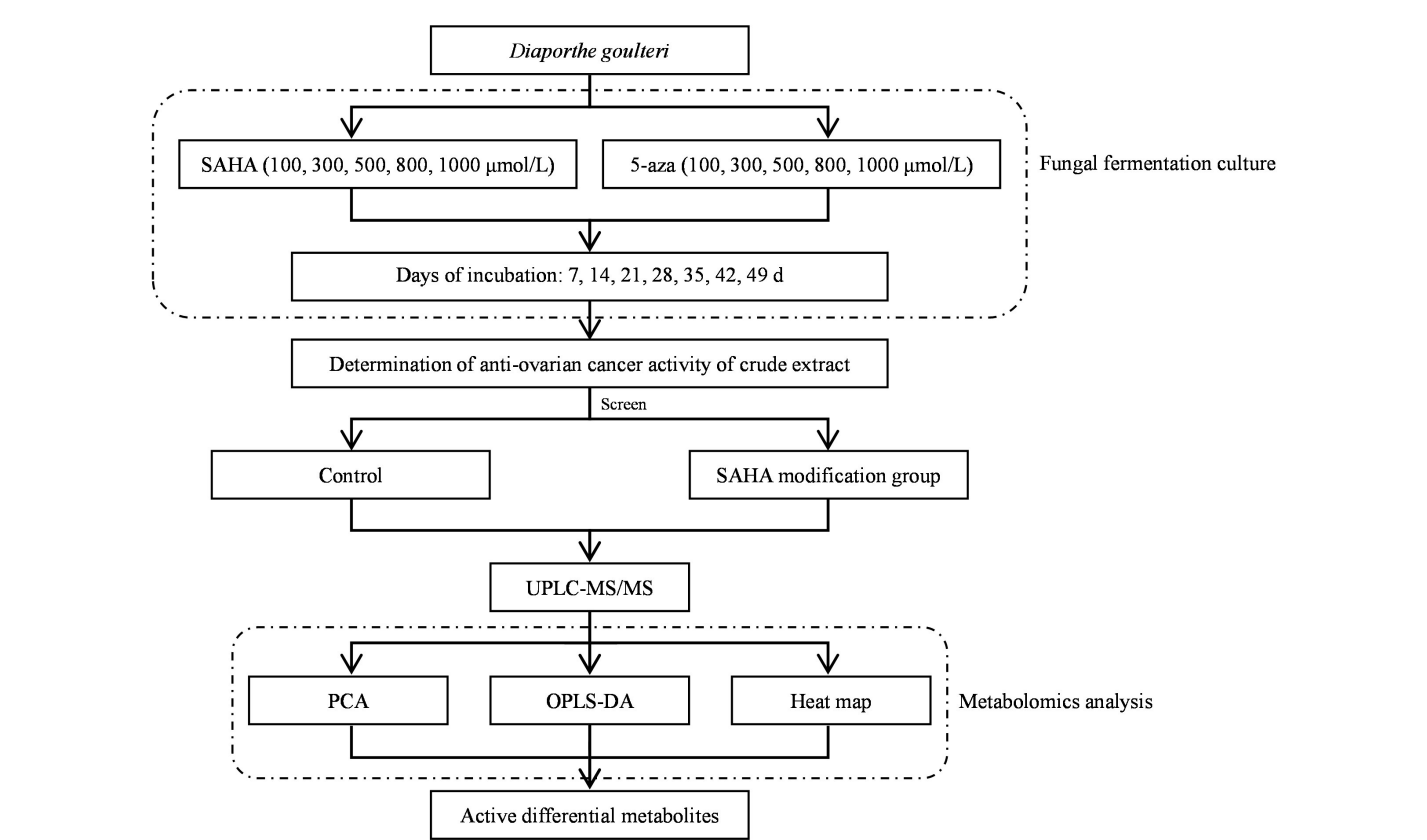
实验流程图

#### 1.2.4 色谱条件

Eclipse Plus C18柱(50 mm×2.1 mm, 1.8 μm);流动相:A相为0.1%甲酸水溶液,B相为乙腈;梯度洗脱程序:0~2 min, 40%B~60%B; 2~5 min, 60%B~80%B; 5~10 min, 80%B~100%B; 10~12 min, 100%B; 12~13 min, 100%B~60%B; 13~15 min, 60%B~40%B。进样量:5 μL;流速:0.3 mL/min;柱温:35 ℃。

#### 1.2.5 质谱条件

离子源为电喷雾离子源(ESI源),正、负离子采集模式,毛细管电离电压3.0 kV,锥孔电压40 V,脱溶剂气(N_2_)温度800 ℃,碰撞气为氩气,脱溶剂气流速800 L/h,锥孔气体流速100 L/h,离子源温度100 ℃,碰撞能量为20~30 V,质谱采集范围为100 ~1500 Da。校正液使用化合物为亮氨酸-脑啡肽(*m/z* 554.2620);软件使用Masslynx V4.1工作站。

#### 1.2.6 数据处理

采用Waters公司的UNIFI信息学系统获取原始数据,进而采用Progenesis QI V2.4软件对原始数据进行峰提取、峰对齐、峰识别和归一化处理等,以QC样品结果作为参考进行标准化处理。基于代谢物的相对分子质量、保留时间和碎片离子等信息,使用Massbank数据库对化合物进行鉴定。采用SIMCA-P 14.1软件对数据进行无监督的主成分分析(principle clustering analysis, PCA),基于有监督的降维方法,采用正交偏最小二乘判别(orthogonal partial least squares-discriminant analysis, OPLS-DA)模型对两组样品进行模式识别分析,从而更好地区分两组样品的代谢组学特征和类别。通过多变量分析中的变量投影重要性(VIP>1)与单变量分析中的*t*检验(*P*<0.05)相结合对差异代谢物进行筛选。

## 2 结果与讨论

### 2.1 活性筛选结果

采用CCK-8法检测84个粗提物对卵巢癌SKOV3细胞的增殖抑制活性。

由表1可以看出,与对照组相比,5-aza调控后所得到的35个粗提物对卵巢癌SKOV3细胞的增殖抑制活性半抑制浓度(IC_50_)值均大于50 μg/mL,初步说明5-aza调控粗提物对SKOV3细胞无抗肿瘤活性。

**表1:** 对照组和5-aza修饰组对SKOV3细胞的半抑制浓度(*n*=3)

Days	Control	Concentrations of 5-aza/(μmol/L)				
		100	300	500	800	1000
7 d	>100	>100	>100	>100	>100	>100
14 d	>100	>100	>100	95.73	>100	90.10
21 d	83.48	76.92	>100	>100	>100	>100
28 d	99.92	88.91	>100	>100	>100	>100
35 d	>100	>100	>100	>100	>100	98.89
42 d	84.52	88.21	>100	>100	>100	75.41
49 d	88.95	76.95	83.83	>100	>100	>100

由表2可以看出,与对照组相比,多个SAHA修饰组抗卵巢癌活性显著提高,分别为发酵7 d的100、300 μmol/L SAHA修饰组,21 d的100、1000 μmol/L SAHA修饰组,35 d的500、800、1000 μmol/L SAHA修饰组,42 d的100、800、1000 μmol/L SAHA修饰组,49 d的300、800 μmol/L SAHA修饰组。其中,抗卵巢癌活性差异最为显著的是发酵培养7 d的100 μmol/L SAHA修饰组,其IC_50_值为0.063 μg/mL,是对照组的277倍。研究表明,若菌株的抗肿瘤活性增强意味着表观遗传修饰剂诱导培养下的菌株新分泌或者增加分泌了具有相应抗肿瘤活性的次级代谢产物,也侧面揭示了菌株在化学表观遗传修饰剂SAHA诱导下活跃的应激反应,由抗肿瘤活性初步评价结果可筛选出合适的SAHA培养体系进行下一步组分差异分析。

**表2:** 对照组和SAHA修饰组对SKOV3 细胞的半抑制浓度(*n*=3)

Days	Control	Concentrations of SAHA/(μmol/L)				
		100	300	500	800	1000
7 d	17.49	0.063	2.329	17.89	23.78	17.87
14 d	35.29	34.49	33.62	31.33	56.64	51.07
21 d	77.71	20.37	84.60	83.25	>100	57.87
28 d	57.86	42.09	56.64	51.26	>100	>100
35 d	71.15	79.07	>100	41.58	45.34	52.53
42 d	38.43	19.14	40.00	>100	15.53	27.25
49 d	48.40	54.54	25.88	>100	27.35	54.86

综上,采用添加100 μmol/L SAHA发酵培养7 d的条件进行扩大发酵,发酵液经提取后进行液相色谱-质谱分析。

### 2.2 UPLC-MS/MS分析

正、负离子模式下对照组与SAHA修饰组的总离子流色谱图见图2,可以看出2组轮廓存在一定差异,但整体相似度较高,为了进一步揭示对照组与SAHA修饰组之间的组分差异,采用代谢组学方法,对反映样本的多个变量进行分析。

**图2:**
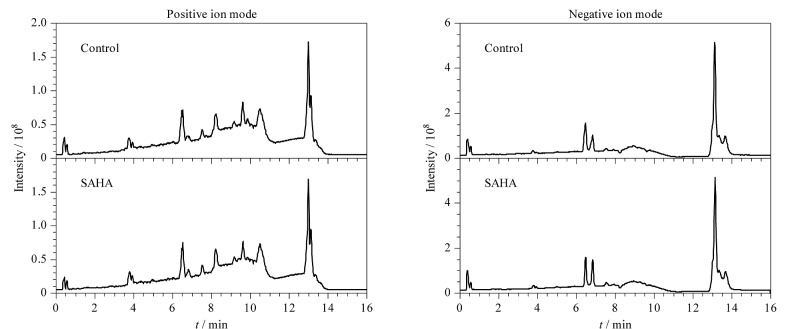
对照组与SAHA修饰组在正负离子模式下的总离子流色谱图

### 2.3 多元统计分析

采用Progenesis QI软件分析正离子和负离子模式下采集得到的数据,分别得到34472和6520个变量,在Massbank数据库中对得到的代谢物进行前体离子与碎片离子的匹配评分,取得分大于39分的化合物,共筛选出132个代谢物用于下一步代谢组学分析。

#### 2.3.1 主成分分析

PCA是一种用于数据降维的统计技术,它通过提取数据中的主要变化方向来减少数据的维度,同时最大限度地保留原始数据的信息。这种方法通常用于识别样本组之间的差异以及评估组内样本的变异性。在本研究中,我们对样本执行了无监督PCA分析,以探究两组样本间的差异,结果见图3。在PCA评分图中观察到,同组的6个样本紧密聚集,表明对照组与SAHA修饰组组内的次生代谢产物具有较高的相似性。两组间比较,它们分别集中在Y轴两侧,表明经SAHA修饰显著改变了真菌的次生代谢产物。其中PC1的贡献率为64.2%, PC2的贡献率为9%, X轴方向模型的累计解释率*R*^2^X=0.732,表明该PCA模型具有较好的拟合度。

**图3:**
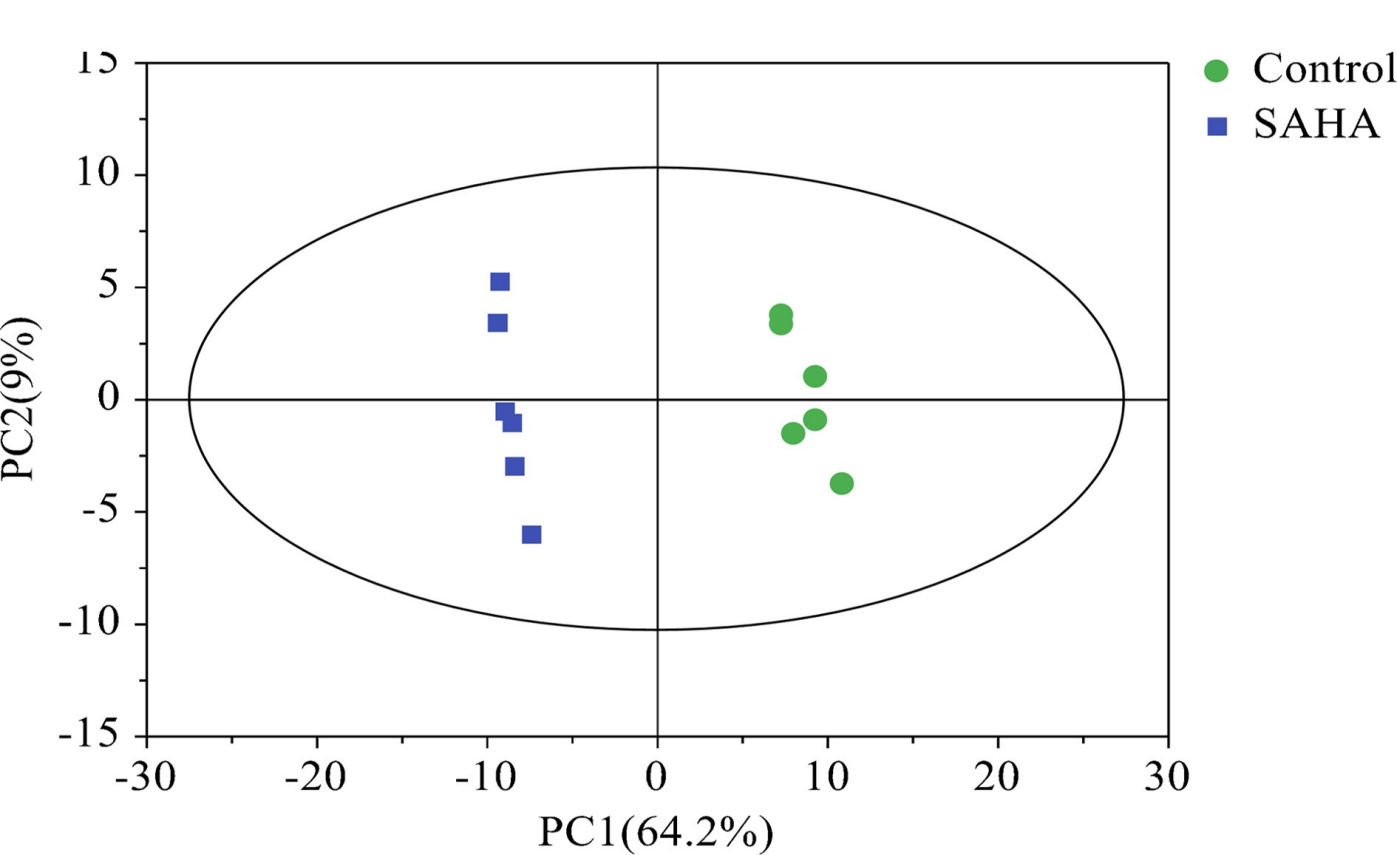
对照组与SAHA修饰组的PCA得分图

#### 2.3.2 正交偏最小二乘判别分析

OPLS-DA是一种结合了偏最小二乘回归(PLS-DA)和正交信号校正(OSC)技术的多变量统计方法,常用于混合物成分的鉴定及区分来源不同或处理方式不同的植物样本。为了深入理解PCA的分析结果,并明确展示两组样本间的相似性和差异性,进而应用了OPLS-DA方法。该模型对两组样品的判别解释能力达99.4% (*R*^2^Y=0. 994),对未知样本的预测能力为98.3% (*Q*^2^=0. 983),均大于0.9,表明该模型参数合理且稳定可靠。OPLS-DA得分图清晰地显示了对照组和SAHA修饰组之间的明显分离,进一步证实了两组样本在代谢产物上的差异,见图4。采用置换测试法对模型进行200次外部交叉验证,见图5, *R*^2^=0.5, *Q*^2^=-1.08,其中*Q*^2^<0,回归线斜率大,与纵轴的截距小,表明模型未过拟合且稳健,具有很好的稳定性和预测性。

**图4:**
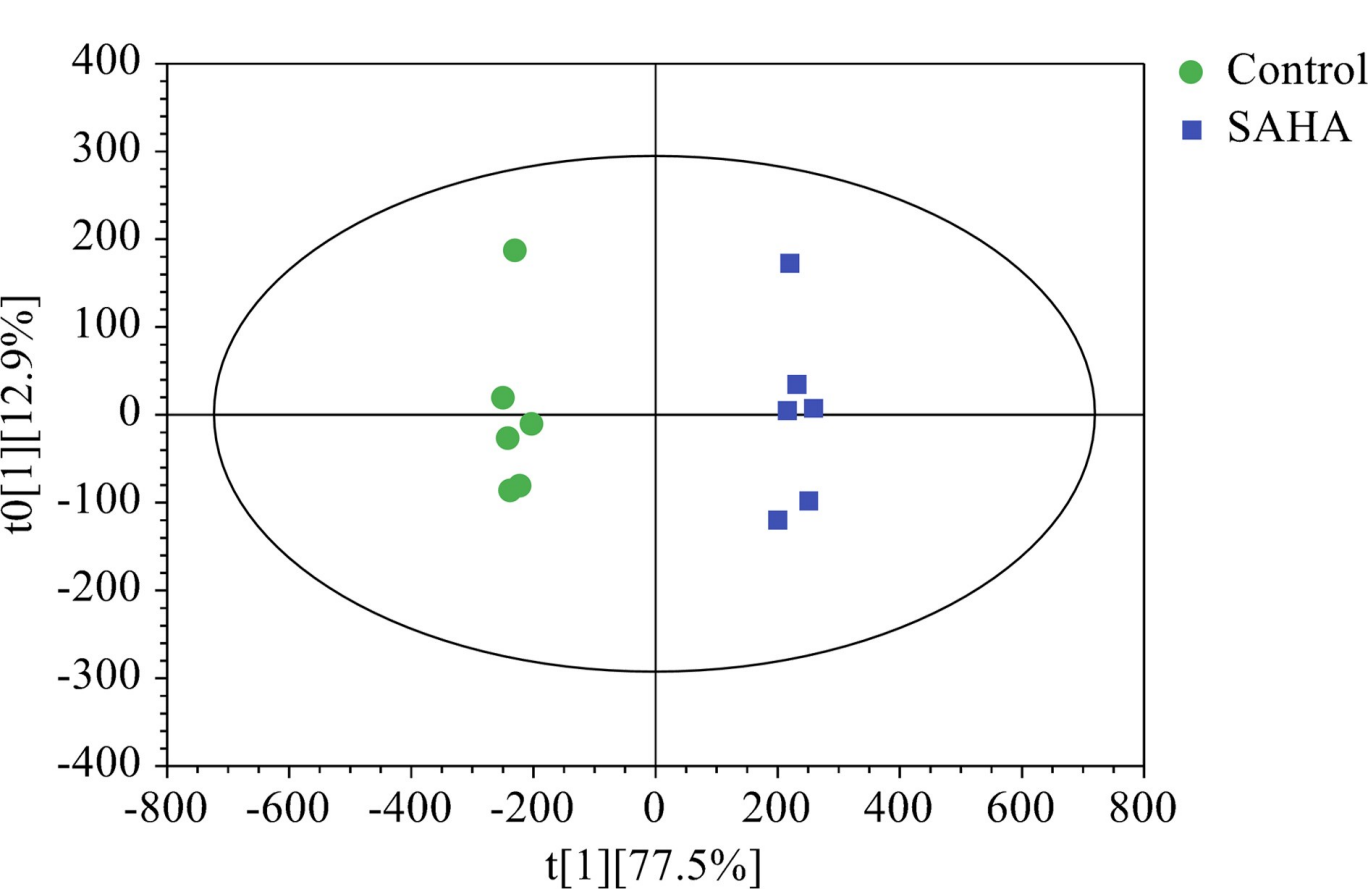
对照组与SAHA修饰组的OPLS-DA得分图

**图5:**
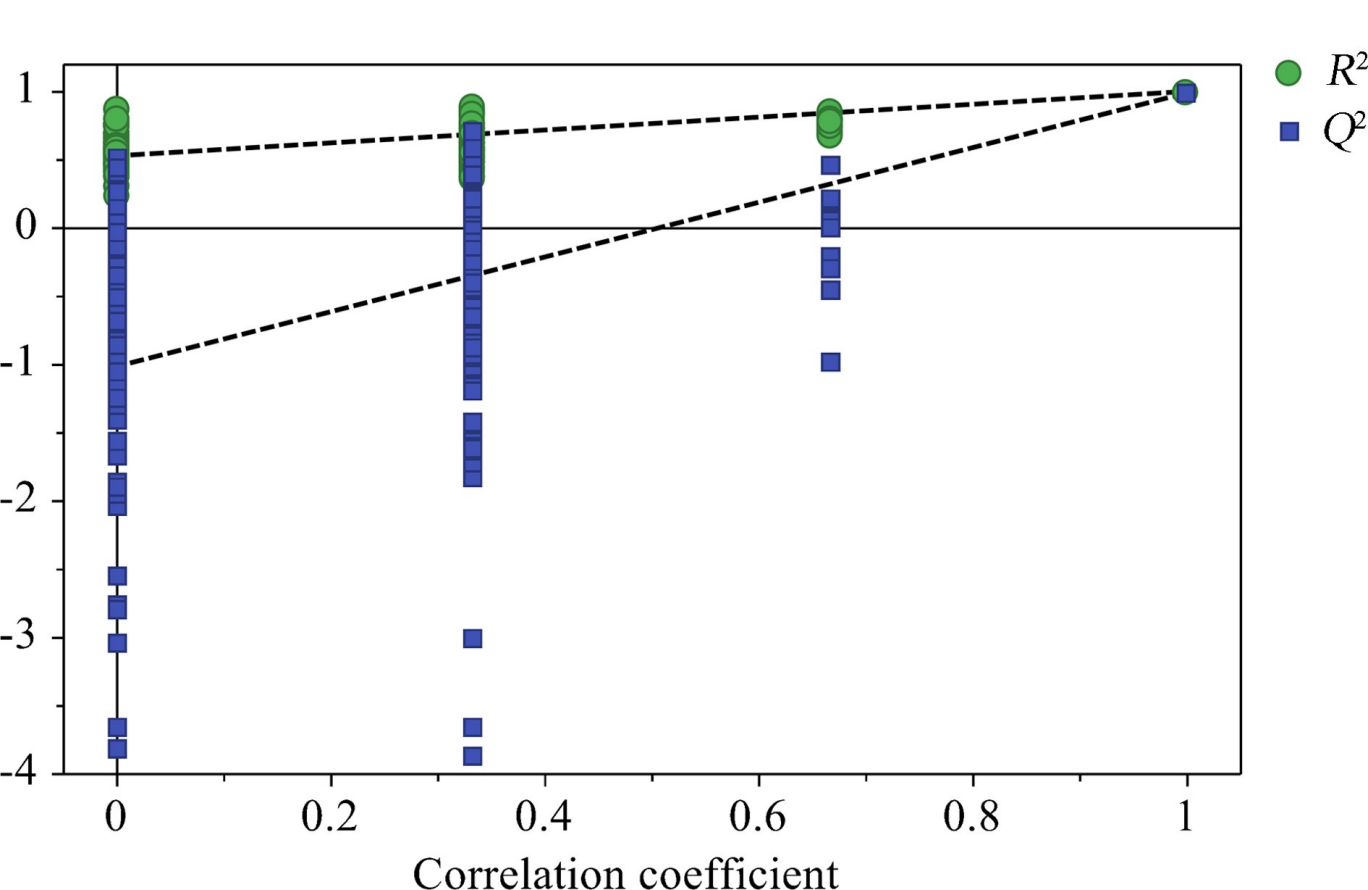
对照组与SAHA修饰组的置换检验图

### 2.4 差异代谢物的筛选

变量重要性投影(variable importance in the projection, VIP)值越大,差异性越强,通常将VIP值大于1的变量视为对模型有显著贡献的变量。根据VIP≥1和*P*<0. 05筛选出15个差异显著代谢物,包括聚酮类化合物3个、氨基酸及衍生物类化合物1个、生物碱类化合物3个,有机酸类化合物1个,其他类化合物7个,见表3。

**表3:** 对照组与修饰组的差异代谢物

No.	Metabolite	*m/z*	*t*_R_/min	Adduct	Ion mode	Formula	Score	VIP score	*P*-value
1	prenderol	155.104	12.81	M+Na	+	C_7_H_16_O_2_	52.1	6.135	****
2	Gly-Val	387.164	9.20	2M+K	+	C_7_H_14_N_2_O_3_	39.6	5.466	**
3	2-ethylcaproic acid	333.228	6.86	2M+FA-H	-	C_8_H_16_O_2_	48.6	4.252	****
4	rubratoxin B	557.143	2.11	M+K	+	C_26_H_30_O_11_	39.0	2.299	****
5	finasteride	407.246	9.26	M+Cl	-	C_23_H_36_N_2_O_2_	54.1	2.247	****
6	6-silaspiro[5.5]undecane	337.274	2.56	2M+H	+	C_10_H_20_Si	39.6	1.895	****
7	1-(2-nitrophenoxy)octane	525.294	12.86	2M+Na	+	C_14_H_21_NO_3_	39.0	1.472	*
8	heptadecene	283.264	9.18	M+FA-H	-	C_17_H_34_	39.0	1.463	****
9	1-pentadecene	255.232	7.54	M+FA-H	-	C_15_H_30_	39.0	1.377	****
10	11-ketoetiocholanolone	343.167	0.72	M+K	+	C_19_H_28_O_3_	39.3	1.323	****
11	3-(1-ethyl-1,3,3-trimethyl-2,3-	534.432	11.44	2M+NH_4_	+	C_18_H_26_O	39.1	1.193	****
	dihydro-1*H*-inden-5-yl)butanaltanal								
12	*N*^2^-benzoylarginine	311.171	2.91	M+CH_3_OH+H	+	C_13_H_18_N_4_O_3_	39.8	1.133	****
13	tabutrex	275.150	6.86	M+FA-H	-	C_12_H_22_O_4_	43.2	1.114	****
14	(3*aR*,6*S*,6*aS*)-6-(4-hydroxy-2-	299.186	0.93	M+FA-H	-	C_15_H_26_O_3_	39.1	1.113	****
	methoxy-2-butanyl)-4,4-dimethyl-								
	hexahydro-1(2*H*)-pentalenone								
15	8-aminoquinoline	333.135	3.07	2M+FA-H	-	C_9_H_8_N_2_	39.1	1.075	****

* *P*<0.05; ** *P*<0.01; **** *P*<0.0001.

### 2.5 差异代谢物的层次聚类分析

对15种差异代谢物进行热图分析,结果见图6,上调物质有10种,占66.67%;下调物质有5种,占33.33%。上调的主要成分为聚酮类化合物,下调的主要成分为生物碱类化合物。

**图6:**
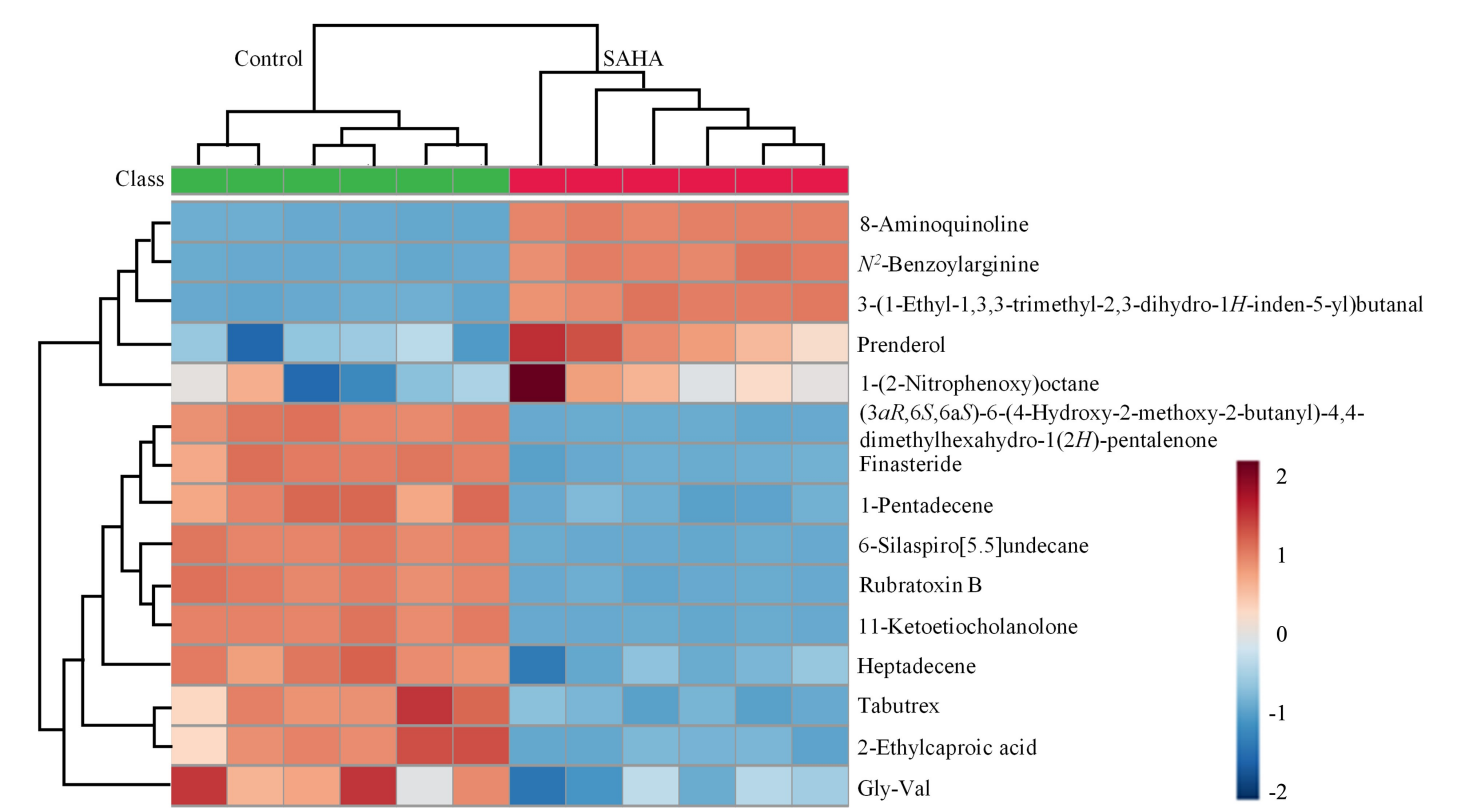
真菌组与SAHA修饰组差异代谢物的相关性分析图

通过OPLS-DA分析得到的S-Plot图进一步验证了对照组与SAHA修饰组组间存在代谢差异,见图7。图7中可视化的所有点全部分布在第一和第三象限,形成了类似S形的分布,越靠近S形末尾两端,表明其在代谢差异中的贡献越大,差异性也越显著。此外,那些聚集在一起的代谢物可能具有相似的生物功能或参与相同的代谢途径。

**图7:**
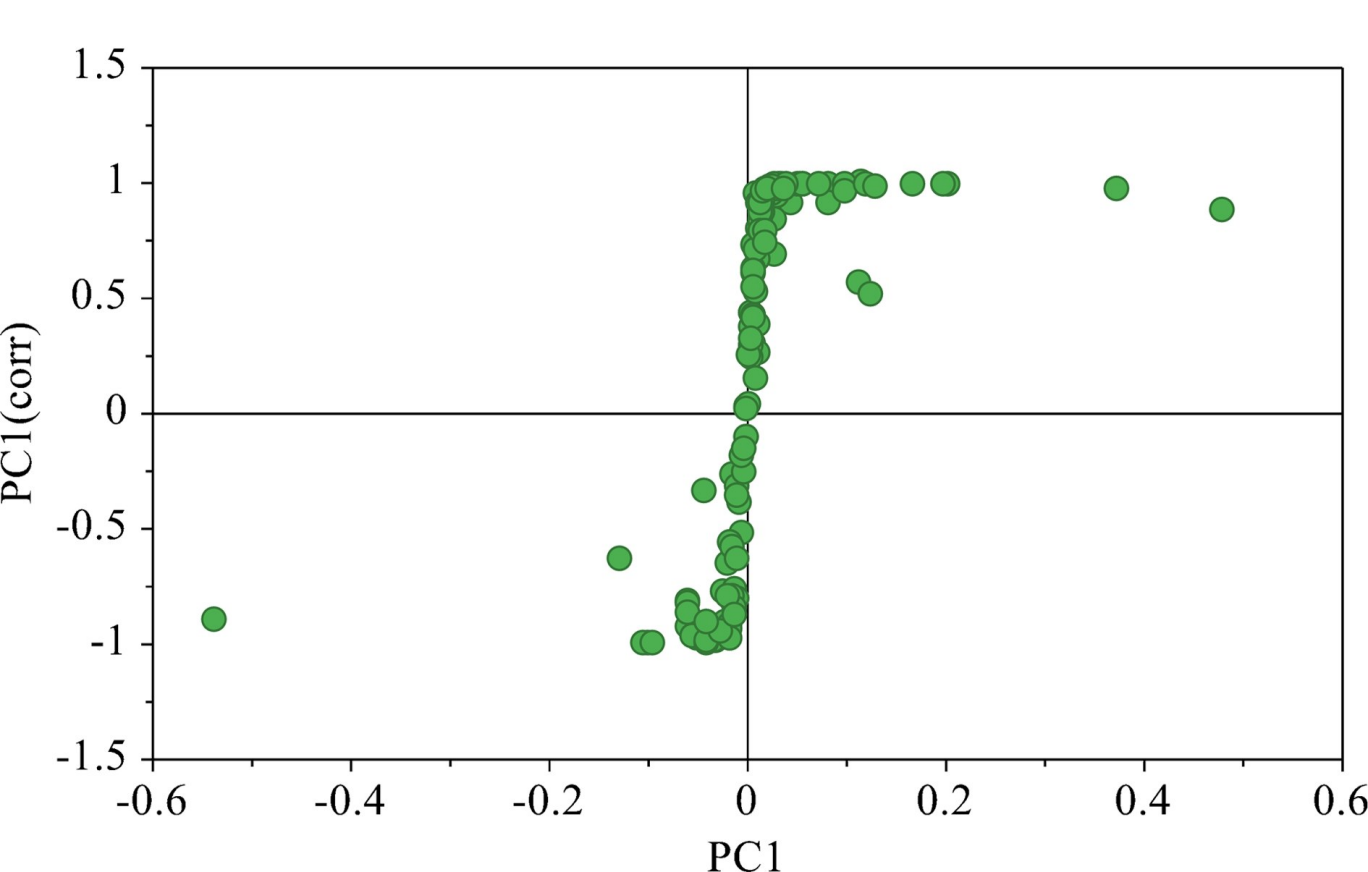
OPLS-DA分析的S-plot图

目前,从老鼠簕内生真菌中分离出的化合物已有上百种,如聚酮、生物碱、简单芳香类化合物、杂萜、醌、大环内酯、核苷以及其他化合物^[18]^。其中,聚酮类化合物为其主要成分。近年来,聚酮类化合物的研究取得了显著进展,特别是在生物合成机制、合成生物学改造以及新化合物的发现方面。聚酮类化合物具有抗菌、抗肿瘤、抗氧化等多种生物活性,某些已被开发为抗癌药物,展现出清除自由基和抗氧化的能力。

经研究发现,在真菌发酵培养基中添加表观遗传修饰剂SAHA可以诱导产生新化合物,其中聚酮类化合物占比居多。例如,He等^[19]^从添加300 μmol/L SAHA的PDB培养基中培养的真菌*Penicillium variabile* HXQ-H-1中分离出7种聚酮,包括4种新的聚酮,即varinectones A和B、沃特曼内酯M(wortmannilactones M)和N。杜林等^[20]^从添加800 μmol/L SAHA的真菌*Daldinia* sp.粗提物中分离得到一种新型氯化五环聚酮Daldinone E。Daldinone E表现出DPPH自由基清除活性,其效力与阳性对照抗坏血酸相当。de Amorim等^[21]^用250 μmol/L SAHA处理内生真菌曲霉菌属*Aspergillus* sp. AST0006的培养物,分离得到两种新的3-(4-氧代吡喃基)-苯并吡喃-2-酮,即aspyranochromenones A和B。

多元统计分析表明对照组与SAHA修饰组的代谢轮廓存在明显差异,为表观遗传修饰剂刺激真菌沉默基因簇,从而产生新化合物提供了理论依据。综合共有差异代谢物聚类分析,发现聚酮类化合物为占比最大的差异代谢物。因此,上调的聚酮类化合物有可能是导致抗卵巢癌活性增强的原因。本研究中采用的基于UPLC-MS/MS技术的非靶向代谢组学分析可以为后续从老鼠簕内生真菌*Diaporthe goulteri*中靶向分离活性化合物提供指导。

## 3 结论

本研究以抗卵巢癌活性为导向,首先筛选出活性好的表观遗传修饰组,继而通过非靶向代谢组学分析,深入探究了表观遗传修饰剂对真菌次生代谢产物的影响,这些次生代谢物不仅在相对含量上存在差异,而且在化合物类型与生物活性上也表现出多样性,这种差异性为发现老鼠簕内生真菌中的新化合物提供了关键线索和研究基础。通过对比对照组与SAHA修饰组的次级代谢产物,我们初步鉴定出的代谢物为后续活性化合物的分离提供了理论依据,这不仅有助于更充分地开发和利用老鼠簕这一红树植物资源,而且为寻找和开发新型天然药物奠定了基础。
